# α4βδ GABA_A_ receptors and tonic inhibitory current during adolescence: effects on mood and synaptic plasticity

**DOI:** 10.3389/fncir.2013.00135

**Published:** 2013-09-03

**Authors:** Sheryl S. Smith

**Affiliations:** Department of Physiology and Pharmacology, SUNY Downstate Medical CenterBrooklyn, NY, USA

**Keywords:** puberty, GABA_A_ receptor, alpha4, delta, anxiety, cognition, tonic current, synaptic plasticity

## Abstract

The onset of puberty is associated with alterations in mood as well as changes in cognitive function, which can be more pronounced in females. Puberty onset in female mice is associated with increased expression of α4βδ γ-amino-butyric acid-A (GABA_A_) receptors (GABARs) in CA1 hippocampus. These receptors, which normally have low expression in this central nervous system (CNS) site, emerge along the apical dendrites as well as on the dendritic spines of pyramidal neurons, adjacent to excitatory synapses where they underlie a tonic inhibition that shunts excitatory current and impairs activation of N-methyl-D-aspartate (NMDA) receptors, the trigger for synaptic plasticity. As would be expected, α4βδ expression at puberty also prevents long-term potentiation (LTP), an *in vitro* model of learning which is a function of network activity, induced by theta burst stimulation of the Schaffer collaterals to the CA1 hippocampus. The expression of these receptors also impairs spatial learning in a hippocampal-dependent task. These impairments are not seen in δ knock-out (−/−) mice, implicating α4βδ GABARs. α4βδ GABARs are also a sensitive target for steroids such as THP ([allo]pregnanolone or 3α-OH-5α[β]-pregnan-20-one), which are dependent upon the polarity of GABAergic current. It is well-known that THP can increase depolarizing current gated by α4βδ GABARs, but more recent data suggest that THP can reduce hyperpolarizing current by accelerating receptor desensitization. At puberty, THP reduces the hyperpolarizing GABAergic current, which removes the shunting inhibition that impairs synaptic plasticity and learning at this time. However, THP, a stress steroid, also increases anxiety, via its action at α4βδ GABARs because it is not seen in δ^−/−^ mice. These findings will be discussed as well as their relevance to changes in mood and cognition at puberty, which can be a critical period for certain types of learning and when anxiety disorders and mood swings can emerge.

## Introduction

Adolescence is a developmental stage when major hormonal and behavioral changes occur. Some reports have characterized adolescence as the end of a critical period for the optimal learning of certain basic tasks, including language acquisition, error detection and spatial memory (Pepin and Dorval, 1986; Johnson and Newport, 1989; Subrahmanyam and Greenfield, 1994; McGivern et al., 2002; Shavalier, 2004). In many cases, the rapid upward trajectory of learning achievement during early development is slowed during the pubertal period (Gur et al., 2012), especially for spatial learning, when gender differences, favoring boys, generally first appear (Kanit et al., 2000; Ardila et al., 2011; Gur et al., 2012). The adolescent period is also known to be a time when emotional changes occur, including mood swings (Buchanan et al., 1992) and increased responses to stress (Susman et al., 1988; Modesti et al., 1994; Lui et al., 2012) as well as the time when anxiety disorders first emerge (Reardon et al., 2009) which, in some cases, continue into adulthood. This review will describe the known changes in populations of extrasynaptic GABA_A_ receptors (GABARs) which occur at puberty and discuss the relevance of these changes in producing behavioral outcomes which limit learning and alter mood during adolescence.

## Critical periods and GABAergic inhibition

Puberty onset has been described as the end of a critical period for optimal learning of certain basic tasks such as learning a second language (Johnson and Newport, 1989). Although there are many factors contributing to the cognitive changes which characterize adolescence, GABAergic inhibition plays an important role in limiting developmental plasticity at this time. This has been shown earlier in development for the visual cortex where the development of GABAergic input marks the end of the critical period of cortical plasticity for ocular dominance (Fagiolini et al., 2004). Because interneurons mature more slowly than excitatory synapses (Huang et al., 1999; Jiang et al., 2005), GABAergic inhibition arrives at a later stage of development when it can slow or even prevent synaptic plasticity (Guo et al., 2013). In fact, positive GABA modulators such as benzodiazepines (BDZs) can alter the timing of the critical period for the visual system (Iwai et al., 2003). This role of GABA in limiting critical periods is widespread throughout development, and is seen for sound localization, taste and olfaction in addition to vision (Hensch, 2004).

## GABA and puberty

**Figure 1:**
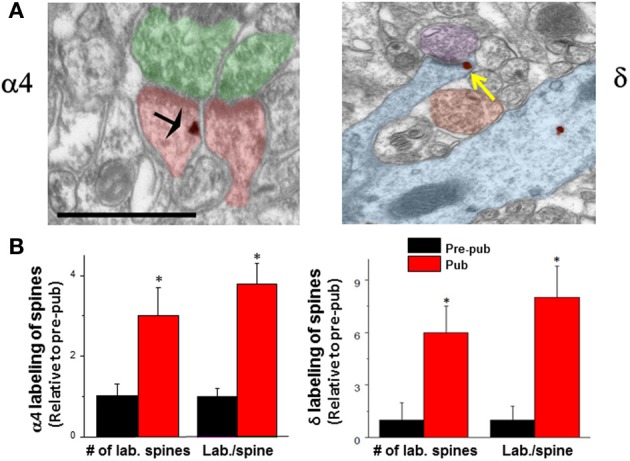
**Expression of α4 and δ GABA_A_ receptor subunit increases on dendritic spines of CA1 hippocampal pyramidal cells at puberty. (A)** α4 (left) and δ (right) silver-intensified immunogold labeling (SIG) occurs along the plasma membrane of spines forming excitatory synapses. α4, black arrow; δ, yellow arrow. Scale, 100 μm. Shafts also exhibit immunoreactivity. **(B)** Averaged data. no of labeled spines (α4, ^*^P < 0.018; δ, ^*^P = 0.002) and labeling per spine (α4, ^*^P < 0.005, δ, ^*^P = 0.00091) increase at puberty (Pub) relative to pre-puberty (Pre-pub). *n* = 50–80 spines [Revised and used with permission (Shen et al., 2010a)].

Several lines of evidence suggest that GABAergic inhibition is greater during adolescence due to both pre- and post-synaptic changes. The number of GABA synapses increases at the time of puberty (Jiang et al., 2005), as does expression of GAD65 (Stork et al., 2000) generating an increase in inhibition at this time. Knock-down of GAD65 has been shown to reduce the tonic inhibitory current (Song et al., 2011) due to a reduction in the concentration of ambient GABA and can affect the timing of critical periods (Katagiri et al., 2007) without altering spontaneous synaptic current (Tian et al., 1999). In addition, tonic inhibition increases in the CA1 hippocampus (Shen et al., 2007), which reduces neuronal excitability by decreasing the input resistance of the neuron. This increase in tonic inhibition is due to increased expression of extrasynaptic α4βδ GABARs on the dendrites of CA1 hippocampal pyramidal cells (Figure 1), which emerge at puberty onset in female mice from almost undetectable levels before puberty (Shen et al., 2007).

## GABARs

GABARs are membrane, pentameric ligand-gated Cl^−^ channels of diverse composition (Olsen and Sieghart, 2009). Although most GABAs are composed of 2α, 2β, and 1γ (Chang et al., 1990), other subytpes exist from a pool of 6α, 3β, 3γ, δ, ε, θ, π, and ρ. These receptors mediate a Cl^−^ conductance, which is inhibitory in most central nervous system (CNS) sites, including hippocampus, after early development (Rivera et al., 1999). GABA current is outward in most CNS sites due to the K+−Cl^−^ co-transporter KCC2 which maintains a Cl^−^ gradient resulting in hyperpolarizing GABAergic current (Payne, 1997). Because the Cl^−^ reversal potential (E_GABA_) is close to the membrane potential (Vm) in many CNS sites, GABA generates a shunting inhibition, regardless of the direction of Cl^−^ current, which in some areas such as dentate gyrus (Staley and Mody, 1992), are in the inward direction (i.e., depolarizing). The shunting properties of GABARs result from the fact that, in these cases, the relatively small driving force for generating Cl^−^ current (Vm − E_GABA_) produces little change in ion flux, but instead primarily reduces the input resistance (*R*) of the neuron because of the opening of Cl^−^ channels. This reduction in input resistance shunts the incoming current (*I*) and reduces its impact in producing a change in membrane voltage, as described by Ohm's Law (Vm = *I* × R). This reduction in input resistance can be independent of the direction of the Cl^−^ current, even though it may be in the depolarizing direction.

Recent reports, however, suggest that the determinants of whether a depolarizing GABAergic tonic current is shunting and inhibitory or excitatory include not only driving force for Cl^−^ current but also the magnitude of the tonic conductance (Song et al., 2011): Smaller GABAergic tonic conductances which are excitatory can be replaced by a shunting inhibition when the conductance is increased by levels of ambient GABA or neurosteroids. Other studies have noted that excitatory versus inhibitory effects of this shunting inhibition also depend upon the precise location of the inhibition as well as the timing of excitatory inputs (Chiang et al., 2012).

### Extrasynaptic GABARs

In addition to synaptic expression, some GABAR sub-types express extrasynaptically where they produce a tonic inhibitory current. These include α_5_β_3_γ_2_ GABARs, localized primarily to CA1 hippocampal pyramidal cells (Wisden et al., 1992; Caraiscos et al., 2004), and α_4_βδ GABARs, localized primarily to dentate gyrus granule cells, thalamic relay nuclei and cortical pyramidal cells (Wisden et al., 1992; Pirker et al., 2000; Stell and Mody, 2002; Stell et al., 2003; Belelli et al., 2005; Chandra et al., 2006) where they generate a tonic inhibitory current (Stell and Mody, 2002). In addition, α_1_βδ and α_1_βγ2 express extrasynaptically on hippocampal interneurons (Semyanov et al., 2003; Glykys et al., 2007; Song et al., 2011) while the α_3_βγ2 does so in basolateral amygdala neurons (Marowsky et al., 2012). The α_6_βδ GABAR, homologous to α_4_βδ, has exclusive expression on granule cells in the cerebellum (Nusser et al., 1998). Other recent studies have shown that α_1_β_2_ expresses extrasynaptically on hippocampal neurons (Sieghart and Sperk, 2002; Mortensen and Smart, 2006).

#### α_4_βδ GABARs

Stoichiometry studies using atomic force microscopy show that subunits within the α_4_βδ GABAR are arranged α_4,_ β, α_4,_ β, and δ, clock-wise, when viewed from the top (Barrera et al., 2008). This receptor has a high sensitivity to GABA (EC_50_ = ~0.5 μ M) (Brown et al., 2002; Sundstrom-Poromaa et al., 2002). Thus, it is well-suited for an extrasynaptic location, where ambient GABA is 100 nM–1 μ M (Wu et al., 2003; Wlodarczyk et al., 2013). Although early studies suggested that these receptors exhibit little desensitization (Bianchi et al., 2002), more recent studies show greater desensitization at both physiological temperature (Bright et al., 2011) and room temperature (Mortensen et al., 2010). Desensitization is rapid in response to rapid exposure to GABA; thus, α4βδ GABARs are likely not activated by transmitter spillover (Bright et al., 2011), but instead generate a steady-state current in response to ambient GABA (Stell and Mody, 2002). A recent study has shown that the extrasyaptic α4βδ GABARs which underlie the tonic current in dentate gyrus granule cells are consititutively active (Wlodarczyk et al., 2013), independent of the low concentrations of ambient GABA found in this region, although increases in the GABA concentration can activate these receptors and increase the tonic current. In the granule cells of the cerebellum, tonic current generated by α6βδ provides a necessary reduction in the high input resistance conferred by the small diameter of the soma. In fact, when these receptors are knocked out, a leak K^+^ channel (TASK) is compensatorily upregulated (Brickley et al., 2001). Similarly, when GABARs containing the α5 subunit are knocked-out, CA1 pyramidal cells compensatorily increase expression of δ-containing GABARs (Glykys and Mody, 2006), while knock-out of δ increases expression of GABARs containing α4 and γ2 subunits in interneurons of the molecular layer of dentate gyrus (Glykys et al., 2008). Taken together, these findings suggest the importance of the tonic inhibitory conductance in neuronal function.

#### Pharmacology of α_4_βδ GABARs

BDZs are typically classified as positive allosteric modulators of most GABARs containing α1-3 or α5 and a γ2 subunit (Olsen and Sieghart, 2009). α_4_βδ GABARs have a unique pharmacological profile because they are insensitive to modulation by BDZs (Wieland et al., 1992; Brown et al., 2002), as are α4βγ2 and α_6_βδ, due to an arginine to histidine substitution at residue 99 of the α4/6 subunit (Wieland et al., 1992), which prevents BDZ binding. In addition, the inclusion of a δ subunit instead of γ2 also renders these receptors BDZ-insensitive (Brown et al., 2002), because α1 and γ2 form the BDZ binding pocket (Buhr and Sigel, 1997); thus α1βδ GABARs are also BDZ-insensitive. GABA acts as a partial agonist at these receptors (Bianchi and Macdonald, 2003), and instead other compounds including gaboxadol [THIP or 4,5,6,7-Tetrahydroisoxazolo(5,4-*c*)pyridin-3-ol hydrochloride], β-alanine and taurine are full agonists at these receptors (Brown et al., 2002; Bianchi and Macdonald, 2003; Jia et al., 2008), such that the response of neurons to these compounds can be used to verify expression of δ-containing GABARs (Shen et al., 2010a,b). α4βδ GABARs are also sensitive targets of steroids such as THP [(allo)pregnanolone or 3α-OH-5α(β)-pregnan-20-one], and THDOC (3α,21-dihydroxy-5α-pregnan-20-one) (Belelli et al., 2002; Brown et al., 2002; Wohlfarth et al., 2002; Bianchi and Macdonald, 2003), which are generally positive modulators of the receptor. These steroids act by increasing receptor efficacy (Bianchi and Macdonald, 2003; Zheleznova et al., 2008). In single channel studies, the steroid THDOC was shown to increase receptor efficacy by adding a third open state of longer duration to the two open states recorded from α4βδ GABARs in the absence of steroid (Wohlfarth et al., 2002). Other studies have shown that, unlike α4β2γ2 GABARs where single channel activity bursts in clusters, recordings from α4βδ GABARs reflect only isolated openings, which have a much lower open probability than other GABARs (Akk et al., 2004; Mortensen et al., 2010). Single channel conductance states of this receptor are similar to α1βγ2, but the mean open time of the highest conductance state is significantly reduced compared to α1βγ2 (Mortensen et al., 2010). In the more commonly expressed α1β2γ2 GABAR, additional studies have been conducted to identify the steroid binding pocket, which extends from the glutamine residue at position 241 in the M1 (transmembrane) segment to asparagine (407) and tyrosine (410) in M4 (Hosie et al., 2006). In this receptor, the steroid THDOC was shown to increase proportion of channels in a long-lived open state (Akk et al., 2010), an effect prevented by mutation of glutamine 241 to serine (Akk et al., 2008), which still permitted steroid potentiation of the receptor. Complete blockade of steroid potentiation of α1β2γ2 was achieved by mutating this glutamine to leucine or tryptophan (Akk et al., 2008).

### Neurosteroids and tonic current

Neurosteroids can increase the tonic current recorded from dentate gyrus granule cells (Stell et al., 2003), although some studies have not observed this effect, due to the rapid metabolism of steroids such as THP and THDOC in this CNS region (Belelli and Herd, 2003). This effect is mediated by α4βδ GABARs because this effect is reduced in δ^−/−^ mice (Stell et al., 2003).

GABARs containing the δ subunit have been shown to be sensitive to low, behaviorally relevant concentrations of alcohol (Sundstrom-Poromaa et al., 2002; Wallner et al., 2003), which enhance the GABAergic tonic inhibitory current (Wei et al., 2004; Glykys et al., 2007). However, several other studies have failed to find effects of low concentrations of ethanol on these receptors (Borghese et al., 2006; Yamashita et al., 2007; Baur et al., 2009) or on tonic current in CNS areas with high expression of δ-containing GABARs (Carta et al., 2004; Borghese et al., 2006; Yamashita et al., 2007). The reason for this discrepancy is not clear, but may be related to the phosphorylation state of the cells recorded, as protein kinase C- δ is required for ethanol effects at δ-containing GABARs (Messing et al., 2007; Choi et al., 2008). Developmental regulation of δ expression may also be a factor in neuronal studies, as δ expression changes across development (Laurie et al., 1992).

## α_4_βδ GABARs and synaptic plasticity at puberty

Expression of α4βδ GABARs is typically quite low on CA1 hippocampal pyramidal cells of adult mice compared to the dentate gyrus granule cell (Peng et al., 2002; Wei et al., 2003), where high expression of these receptors yields a tonic current that is 5 to 6-fold greater than that measured in CA1 pyramidal cells assessed by comparing current amplitude in wild-type versus mice which lack δ expression (δ^−/−^) (Glykys et al., 2008). However, there are marked increases in expression of this receptor on CA1 pyramidal cells at puberty from these nearly undetectable levels noted before puberty (Shen et al., 2007). The increase in α4βδ GABAR expression on CA1 hippocampal pyramidal cells of female mice at puberty is localized both to the dendritic shaft as well as the dendritic spine (Figure 1) adjacent to asymmetric, excitatory synapses (Shen et al., 2010a). This unique location suggests a role for these receptors in regulating cognition during adolescence, because the dendritic spine is the CNS site for induction of synaptic plasticity (Nevian and Sakmann, 2006). Although GABAergic interneurons target dendritic spines in cortical circuits (Kubota et al., 2007), GABAergic input does not do so in CA1 hippocampus, where GABARs, either synaptic or extrasynaptic are not typically found. In adult CA1 hippocampus, GABARs localize to the soma (80%) or the dendritic shaft (20%) (Megias et al., 2001), where they can either be sub-synaptic or extrasynaptic [predominantly α5β3γ2 (Brunig et al., 2002)]. Only during the pubertal period (~post-natal day 35–44) do extrasynaptic α4βδ GABARs increase to significant levels of expression (Shen et al., 2010a,b): The proportion of spines which are immunolabeled with α4 and δ is increased by 3 to 6-fold, respectively, while the immunoreactivity per spine is increased 4 to 8-fold, respectively, as determined with silver-intensified immunogold (SIG) labeling and electron microscopy in the proximal stratum radiatum (Figure 1). Recent estimates suggest that 25% of spines may express α4βδ GABARs (Aoki et al., 2012). Similar increases in immunolabeling are seen on the dendritic shaft. Pubertal expression of α4βδ GABARs in these dendritic compartments persists for a period of about 10 d, and is reduced significantly by about post-natal day 44 (Aoki et al., 2012).

Although it is not possible to know whether this receptor is increased at puberty in humans, there is indirect evidence suggested by the reduced sensitivity of adolescents to the sedative effect of BDZs such as midazolam (Massanari et al., 1997), which would be consistent with increased expression of α4βδ, a BDZ-insensitive GABAR (Wisden et al., 1991; Wafford et al., 1996). There is also an increased incidence of paradoxical anxiety reactions to BDZs in adolescents (Massanari et al., 1997), which is consistent with increased expression of α4βδ on principal neurons (but not on interneurons, where BDZs could preferentially disinhibit the network). Although not definitive, this evidence is at least consistent with the predicted pharmacology if α4βδ GABARs were increased during adolescence in humans.

## Physiological consequences of α_4_βδ GABAR expression

**Figure 2:**
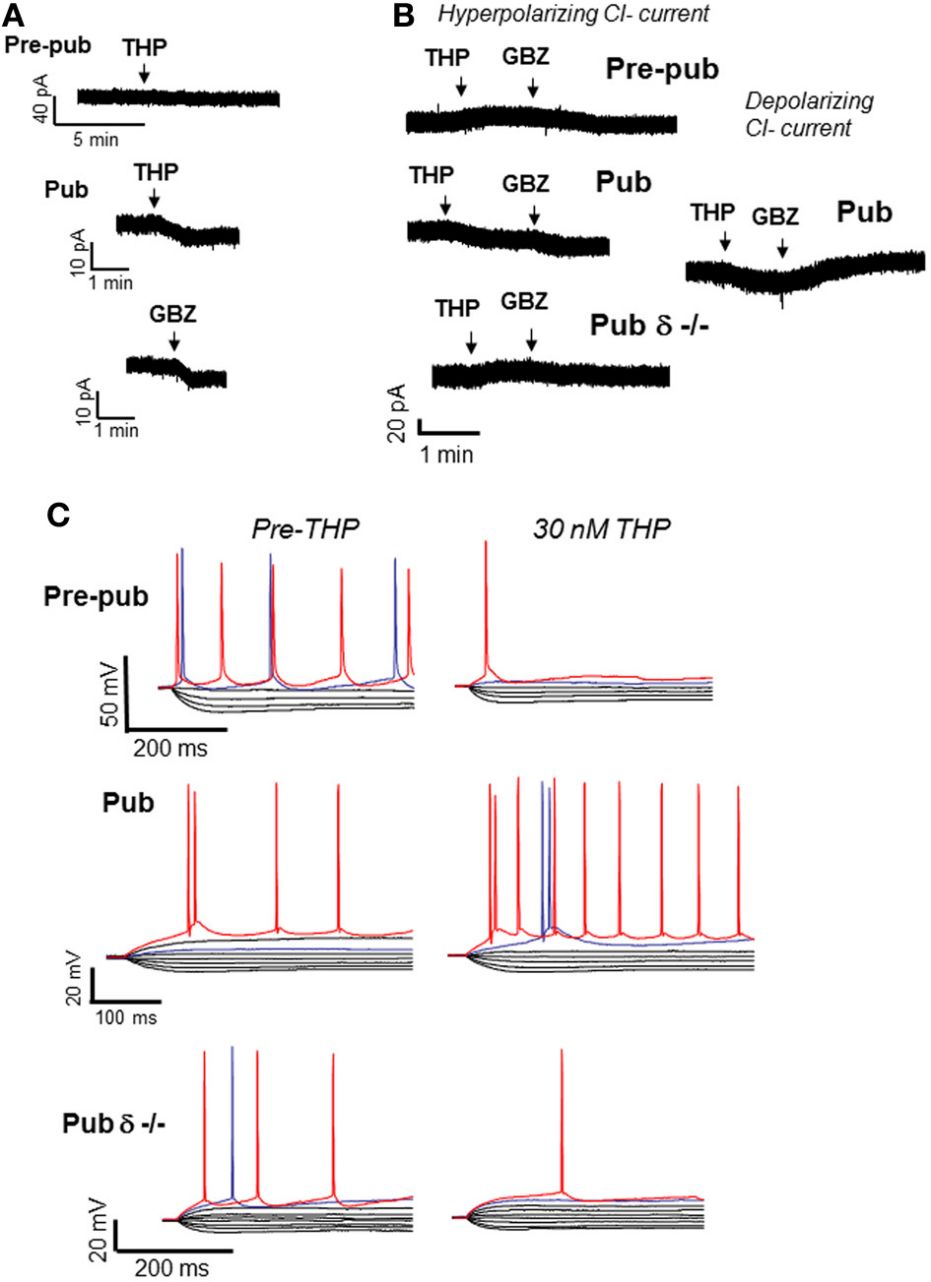
**Effects of THP on CA1 hippocampal pyramidal cells at puberty**. At the onset of puberty, THP reduces the tonic GABAergic current and increases excitability of CA1 pyramidal cells in the slice. **(A)** THP (30 nM) effects on the tonic current recorded with gramicidin perforated patch techniques to maintain the internal Cl^−^ milieu. 1 μ M TTX, 1 μ M GABA, and 2 mM kynurenic acid were added to isolate the GABAergic post-synaptic component. L-655, 708, CGP55845, and TEA were also added to block α5 and GABA_B_ receptors and K+ channels, respectively. Pre-pub, pre-pubertal; Pub, pubertal; GBZ, gabazine, a GABA antagonist (presented for comparison). THP reduces the current in Pub slices. (Representative of 5 cells/group) **(B)** Left panel, Hyperpolarizing current recorded from CA1 hippocampal pyramidal cells in the slice using whole cell patch clamp techniques (ECl = −70 mV, −50 mV holding potential; pipette solution, K-gluconate; bath, 200 nM gabazine to block synaptic current and 2 mM kynurenic acid to block excitatory current). Right panel, effects of THP on the depolarizing tonic current at puberty (ECl = −30 mV, pipet solution, CsCl). THP reduces hyperpolarizing current in wild-type but not δ^−/−^ mice, but potentiates depolarizing current. (Representative of 8–12 cells/group) **(C)** Whole-cell current-clamp recordings reveal voltage responses recorded in response to increasing 0.3-nA current injection (initial current −1 nA). (The THP trace lacks the 800-pA current trace for ease of comparison.) THP lowers the current threshold for spiking of pyramidal cells at the onset of puberty in wild-type but not δ^−/−^ mice. Red trace, equivalent current injection, threshold for the less excitable state. Blue trace, equivalent current injection, threshold for the more excitable state. (Representative of 7–8 cells/group) [Revised and used with permission (Shen et al., 2007)].

**Figure 3:**
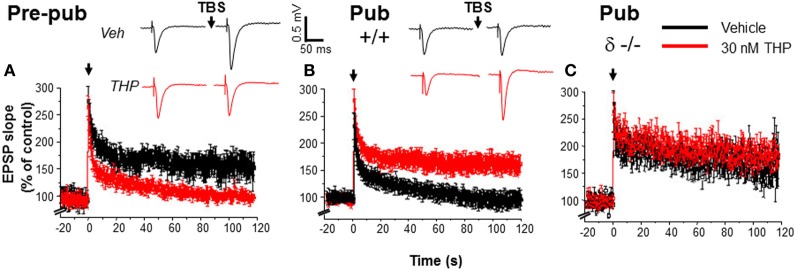
**Impaired induction of LTP at puberty: reversal by the stress steroid THP and δ knock-out**. Theta burst stimulation (TBS—0 time) induced LTP (black) before puberty Pre-pub, **(A)** but not in the pubertal Pub, **(B)** CA1 hippocampus. THP (red, 30 nM) permitted LTP induction at puberty. (Inset, Representative field EPSPs. TBS, arrow. Scale, 0.5 mV, 50 ms.) **(C)**. LTP, Pubertal δ^−/−^, where TBS induced LTP. *n* = 6–9/group. [Used with permission (Shen et al., 2010a)].

Functional expression of α4βδ at puberty was verified by the robust response of CA1 hippocampal pyramidal cells to the GABA agonist gaboxadol at a 100 nM concentration (Shen et al., 2010a), selective for α4βδ GABARs (Brown et al., 2002; Meera et al., 2011). In contrast, gaboxadol produces a negligible response in pre-pubertal CA1 hippocampus. This increase in α4βδ GABAR expression at puberty is associated with a number of predictable outcomes, including a decrease in the input resistance and an increase in the threshold for action potential activation in response to injection of depolarizing current (Figure 2). In addition, activation of NMDA receptors is impaired (Shen et al., 2010a) most likely due to the shunting inhibition produced by these receptors which would reduce the depolarization necessary for Mg+ unblock of the receptor (Herron et al., 1986). Consequently, induction of long-term potentiation (LTP), an *in vitro* model of learning, produced by stimulation of the Schaffer collaterals to CA1 hippocampus with theta burst stimulation (TBS), is impaired (Figure 3) (Shen et al., 2010a). This deficit in synaptic plasticity is prevented with total blockade of GABARs (120 μ M SR95531) (Stell and Mody, 2002) or with the use of the δ^−/−^ mouse. Thus, these data suggest that α4βδ GABARs which emerge at puberty impair synaptic plasticity during adolescence. In contrast, LTP induction is robust in the hippocampus of pre-pubertal mice. Surprisingly, selective blockade of synaptic GABARs (200 nM SR95531) (Stell and Mody, 2002) does not facilitate LTP induction at puberty, suggesting that the deficit in synaptic plasticity is due to the extrasynaptic GABAR population exclusively (Shen et al., 2010a). Extrasynaptic α5β3γ2 GABARs also play a role in limiting synaptic plasticity induced by low frequency stimulation in adults, where synaptic GABARs are not a factor (Martin et al., 2010). In dentate gyrus, which has high expression of α4βδ GABARs that generate a robust tonic inhibition (Wei et al., 2003; Glykys et al., 2008), tonic inhibition plays a major role in modulating LTP in adult hippocampus, with greater effects than noted in the CA1 hippocampus (Arima-Yoshida et al., 2011). However, high frequency stimulation also differentially increases synaptic inhibition more than synaptic excitation in adult hippocampus, which suggests that synaptic inhibitory current may play a role in altering synaptic plasticity in the adult although this has not been definitively demonstrated (Arima-Yoshida et al., 2011).

Earlier studies suggested that LTP induction is impaired in adolescence due to an increase in GABAergic inhibition (Meredith et al., 2003), although puberty onset and α4βδ were not identified in this study. In contrast, the function of NMDARs does not appear to be compromised at puberty because activation of NMDA current is robust in the δ^−/−^ or after total GABAR blockade (Shen et al., 2010a). Therefore, the deficit in synaptic plasticity at puberty appears to be due to increases in shunting inhibition mediated by α4βδ GABARs.

## Pubertal expression of α_4_βδ GABARs and spatial learning

**Figure 4:**
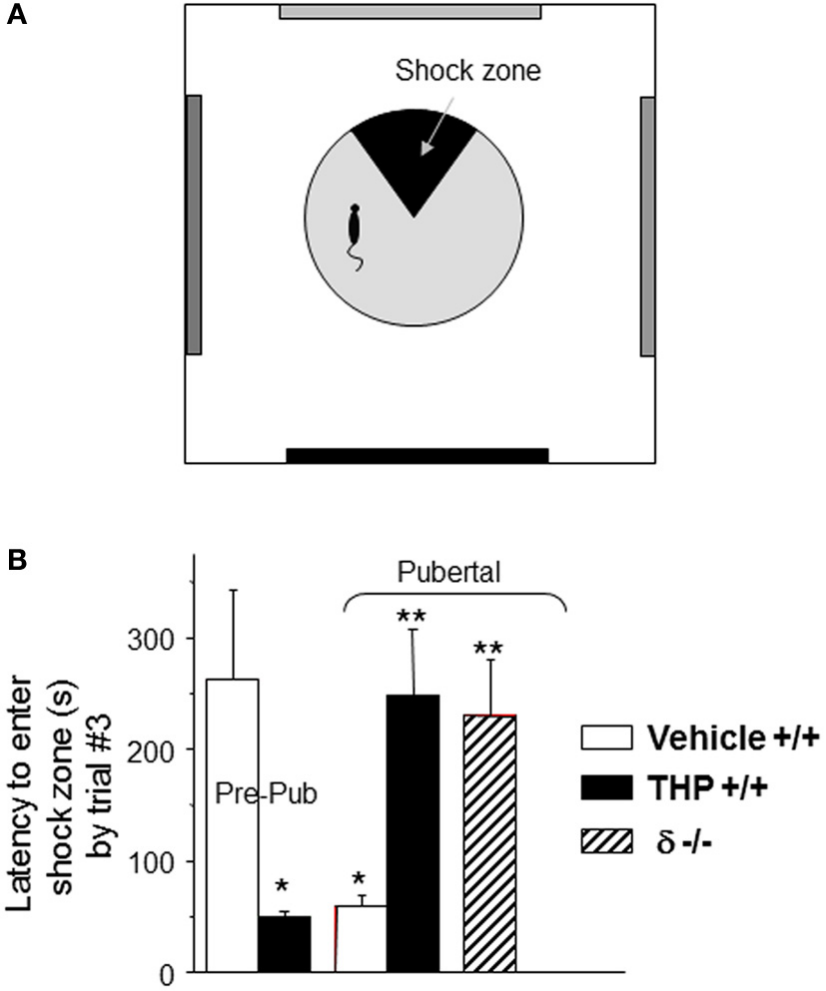
**Impaired spatial learning of pubertal mice: reversal by the stress steroid THP and δ knock-out. (A)** Spatial learning platform (shock zone, black sector). The platform rotates, but the shock zone is stationary, requiring the animal to move to actively avoid the shock. **(B)** Latency to first entry of the shock zone, a measure of learning. Pre-pub mice attained the longest entry times. (*n* = 6–9 mice/group) ^*^P < 0.05 vs. pre-pub; ^**^P < 0.05 vs. pub +/+ vehicle. [Revised and used with permission (Shen et al., 2007)].

Because α4βδ GABARs localize to dendritic spines of pyramidal cells in CA1 hippocampus, the behavioral outcome has been tested in a spatial learning task. The CA1 hippocampus plays a critical role in spatial learning (Burgess et al., 2002; Bannerman et al., 2004; Pastalkova et al., 2006), where selective deletion of NMDARs in this region impairs the spatial and contextual forms of memory without affecting the temporal aspects of memory formation (Tsien et al., 1996; Place et al., 2012); thus, a hippocampal-dependent task (Cimadevilla et al., 2001) was selected to demonstrate the role of these receptors in spatial learning. To this end, an active place avoidance task has been employed which requires that the animal avoid a mild footshock (<0.2 mA) sub-threshold for release of stress steroids (Friedman et al., 1967), which suggests that this is a relatively unstressful task compared to other animal models of learning (Harrison et al., 2009). For each trial, the latency to enter the avoidance sector during rotation of the platform is a measure of learning. Performance on this task is well-correlated with, and depends upon, successful induction of LTP (Pastalkova et al., 2006). Pubertal mice show impaired spatial learning on this task compared to pre-pubertal mice: they show faster latencies to re-enter the avoidance zone during learning trials and fail to reach criterion (120 s latency to enter the shock sector) after 7 trials (Figure 4). In contrast, pre-pubertal mice reach criterion in 2.5 trials (with a mean latency of 275 s) (Shen et al., 2010b). Potential non-specific effects of other sensorimotor/behavioral outcomes were ruled out in this study because general locomotor activity was not altered (assessed as path length). In addition, the number of shocks/entry was not different between groups suggesting that the shock is equally aversive for all animals and that they are equally able to escape (which would include sensorimotor, motivational and attentional parameters). This learning deficit is not seen in pubertal δ^−/−^ mice, suggesting that it is the increase in α4βδ GABAR expression at puberty which produces these deficits in spatial learning (Shen et al., 2010a). This is most likely due to the shunting inhibition produced by these receptors which impairs activation of hippocampal NMDARs.

## α_4_βδ GABA_A_ receptors: effects on plasticity and learning in adult, male mice

Because α4βδ GABARs have high expression in the dentate gyrus, recent studies have focused on examining effects of α4 or δ knock-out on learning tasks that are relevant for this area, including trace and conditional fear conditioning, recognition memory and contextual discrimination memory. Although the dentate gyrus circuitry plays a role in the formation of these memories, the specific effect is complex and the outcome of α4βδ knock-out depends on the task examined. Both α4 and δ knock-out enhance trace and conditional fear conditioning (Wiltgen et al., 2005; Moore et al., 2010). However, δ knock-out impairs recognition memory and contextual discrimination memory, suggesting that these receptors facilitate these types of plasticity (Whissell et al., 2013). Intriguingly, this study showed that α4βδ GABARs facilitate neurogenesis in this area (Whissell et al., 2013), which is a likely mechanism for their facilitating effect on learning. As in the previous study, fear memory was enhanced by δ knock-out, but fear memory extinction was impaired, suggesting that the mechanisms for acquisition and extinction of fear memories require different mechanisms.

Another recent study has suggested that α4βδ GABARs may also act presynaptically to regulate neurotransmitter release from mossy fiber afferents to CA3 pyramidal cells (Ruiz et al., 2010). In this case, low concentrations of the neurosteroid THDOC facilitate glutamate release, while the GABA antagonist SR95531 reduces glutamate release and impairs induction of LTP at CA3 synapses. Again, this is an additional example of a case where α4βδ GABARs facilitate synaptic plasticity, suggesting that their effect depends not only on the age of the animal but also on the circuit involved. There are limitations in our understanding of the exact impact of these receptors, however, because it is not possible to localize receptor deletion to specific sites within specific brain areas. Until that becomes possible, our understanding of their impact is restricted to broader regions of the CNS.

The role of the dentate gyrus in mediating the changes in cognition at puberty is not known. Changes in receptor expression have not been quantified in this region across pubertal stages. However, it is likely that effects of THP would enhance recognition memory and contextual discrimination memory at puberty via its ability to potentiate tonic inhibition mediated by α4βδ GABARs expressed on dentate gyrus granule cells, where it would likely facilitate neurogenesis. Thus, this is an important topic for future studies.

## Positive modulators of GABA_A_ receptors and synaptic plasticity

It is well known that positive modulators of the GABAR impair synaptic plasticity and learning, as reported in both rodents and humans. Benzodiazepine tranquilizers are amnestic (Veselis et al., 2009), as are certain anesthetics such as propofol (Veselis et al., 2009) and isoflurane (Saab et al., 2010). Both alcohol and the neurosteroid THP impair spatial learning on the Morris Water Maze (Matthews et al., 2002). In adult CA1 hippocampus, α5β3γ2 GABARs have high expression extrasynaptically where they localize to the dendritic shaft and to the base of the dendritic spine (Brunig et al., 2002). Both knock-out and knock-down of this receptor, with the use of a selective inverse agonist, prevents impairments in learning following administration of the anesthetic etomidate (Cheng et al., 2006). These procedures also enhance fear conditioning (Collinson et al., 2002; Crestani et al., 2002; Chambers et al., 2003). Positive GABA modulators also impair synaptic plasticity in *in vitro* models, including LTP: The degree of synaptic potentiation is significantly decreased by alcohol, THP and propofol (Izumi et al., 2005; Nagashima et al., 2005; Ma et al., 2005; Tokuda et al., 2011), suggesting that GABA inhibition plays an important role in limiting synaptic plasticity in the hippocampus. In contrast, α5 knock out reduces the threshold for frequency-dependent induction of LTP (Martin et al., 2010).

## THP and the response to stress

THP is a GABA-modulatory metabolite of progesterone, and is produced both by the ovary and adrenal gland (Mellon and Vaudry, 2001), which increase release of THP before puberty onset (Mannan and O'Shaughnessy, 1988; Fadalti et al., 1999; Shen et al., 2007). Fluctuations in circulating levels of THP occur across the ovarian cycle, reaching peak levels on the afternoon of proestrus and day of diestrus 1, as well as during pregnancy (Palumbo et al., 1995; Concas et al., 1998). In addition, THP can be produced *de novo* in the brain from cholesterol via side chain cleavage enzyme (Compagnone and Mellon, 2000) in several CNS sites, including the CA1 hippocampal pyramidal cell (Agis-Balboa et al., 2006). Circulating and/or CNS levels of this steroid increase before puberty, but decline to low levels at the onset of puberty, (Fadalti et al., 1999; McCartney et al., 2007; Shen et al., 2007). Unlike most steroids, THP has no known effect at classic nuclear steroid receptors, but instead is a modulator of the GABAR (Smith et al., 2007).

In rodents, circulating levels of THP increase by up to 20-fold after 45 min of restraint stress and other forms of stress, such as CO_2_ inhalation (Purdy et al., 1991; Higashi et al., 2005; Mukai et al., 2008) when decreases in anxiety are observed (Barbaccia et al., 2001). Similarly, in humans, circulating levels of this steroid increase after sustained stress associated with performance (Droogleever Fortuyn et al., 2004; Girdler et al., 2006). Thus, THP is one factor which is part of the stress response, and because it typically acts as an anxiolytic (Bitran et al., 1999), it would be expected to mitigate the anxiety reaction to stress in adults.

## Polarity-dependent actions of THP at α_4_βδ GABARs

**Figure 5:**
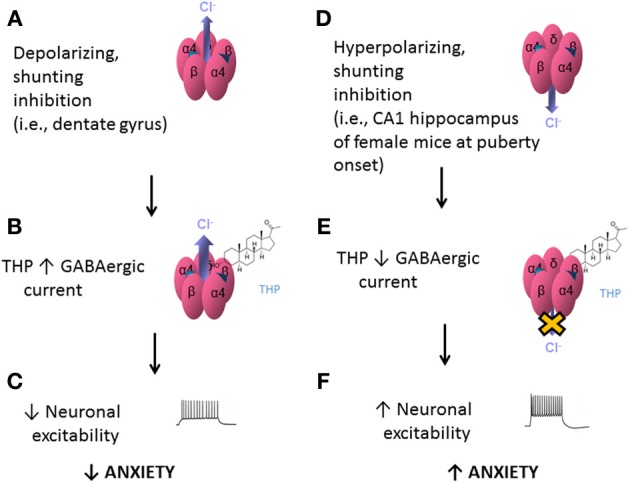
**Effects of THP on α4βδ GABARs: summary diagram**. Effects of the neurosteroid THP at α4βδ GABARs are dependent upon the polarity of GABAergic current. α4βδ GABARs are depicted with inward, depolarizing current (outward Cl^−^ flux, A) or outward, hyperpolarizing current (inward Cl^−^ flux, D). **(A)** When the Cl^−^ current generated by these receptors is inward, as would be found in dentate gyrus granule cells, THP potentiates this shunting inhibition **(B)** However, when the Cl^−^ current generated by these receptors is outward, as is found in CA1 hippocampal pyramidal cells at puberty **(D)**, THP reduces this current **(E)**, via acceleration of the rate and increases in the extent of desensitization (Shen et al., 2007). This differential effect of the steroid, which is dependent upon the direction of Cl^−^ current through the GABA channel, would have different outcomes at the level of the circuit and behavior: When current generated by α4βδ GABARs is a depolarizing, shunting inhibition **(A–C)**, THP would decrease neuronal excitability in limbic structures and decrease anxiety **(C)**. In contrast, when the current generated by α4βδ GABARs is hyperpolarizing **(D–F)**, as observed at puberty, THP increases neuronal excitability and anxiety **(F)**.

The effects of THP at α_4_βδ GABARs appear to be dependent upon the direction of the Cl^−^ current in the GABA channel. When GABA-generated current is in the depolarizing direction (inward current), THP and related steroids *increase* current recorded from recombinant α_4_βδ GABARs (Belelli et al., 2002; Wohlfarth et al., 2002; Bianchi and Macdonald, 2003) (Figures 5A,B). In contrast, when GABA-generated current is in the hyperpolarizing direction (outward current), THP *decreases* current recorded from recombinant α_4_βδ GABARs (Shen et al., 2007) (Figures 5D,E). The first outcome is relevant for the dentate gyrus granule cell (Figures 5A–C), where GABA generates a depolarizing, shunting inhibition (Staley and Mody, 1992), and THP increases the tonic, inhibitory current (Staley and Mody, 1992) and reduces neuronal excitability (Stell et al., 2003). This is consistent with the typical effect of THP to reduce anxiety (Bitran et al., 1999). The second outcome is relevant for CA1 hippocampal pyramidal neurons which generate a hyperpolarizing current generated by α_4_βδ GABARs which express during adolescence (Shen et al., 2007). At this time THP reduces the tonic inhibitory current and increases neuronal excitability (Shen et al., 2007) (Figures 5D–F). Consistent with this novel excitatory effect, THP now increases anxiety, in contrast to its typical anxiety-reducing effect (Shen et al., 2007).

**Figure 6:**
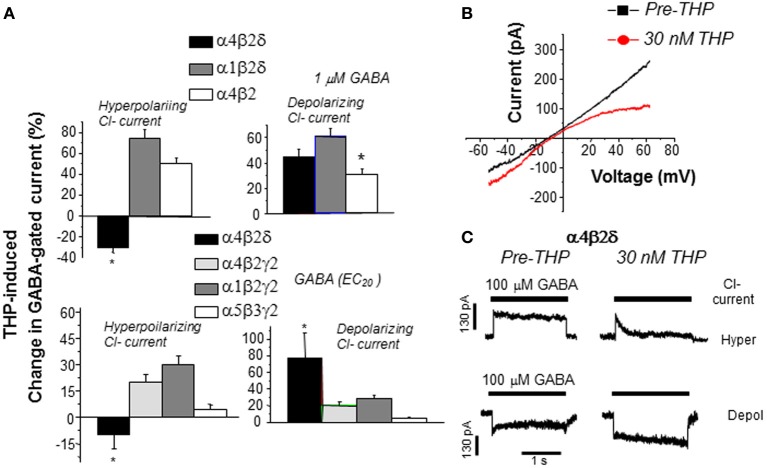
**THP exerts polarity-dependent effects at α4βδ GABA_A_ receptors**. THP, a neurosteroid, decreases hyperpolarizing current gated by α4βδ GABA_A_ receptors expressed in HEK-293 cells (recorded with whole cell voltage clamp techniques). **(A)** THP effects on hyperpolarizing and depolarizing currents in response to 1 μ M GABA (upper panel) or the GABA EC_20_ (lower panel; α4β2δ, 0.1 μ M; α4β2γ2, 5 μ M; α1β2γ2, 10 μ M; α5β3γ2, 5 μ M) from 6–7 cells for each group (Mean ± SEM; ^*^P = 0.05 vs. the other receptor subtypes). *n* = 6–8 cells/group. **(B)** Effects of THP (30 nM) on current in response to a voltage ramp over 400 ms where the ECl = 0 mV. THP increases depolarizing current (voltage < 0 mV) but decreases hyperpolarizing current (voltage > 0 mV). (Leak-subtracted current is presented as the average of three traces). *n* = 5–6 cells/group. **(C)** Effects of THP on desensitization of hyperpolarizing (upper traces) and depolarizing (lower traces) current at α4βδ receptors (representative of 6 cells per group). THP accelerates desensitization of the hyperpolarizing current. [Used with permission (Shen et al., 2007)].

THP's novel effect to reduce GABA current generated by α_4_βδ GABARs are seen in recombinant receptors where either the ion gradient or holding potential are varied, and occur in the absence of alterations in the reversal potential, which suggests that other conductances are not involved (Figure 6) (Shen et al., 2007). THP-induced decreases in hyperpolarizing current recorded from α_4_βδ GABARs were shown to be due to acceleration in the rate and extent of receptor desensitization (Shen et al., 2007). This conclusion is consistent with findings from Macdonald and colleagues who have shown that α_4_βδ GABARs desensitize more rapidly when the current is in the hyperpolarizing direction (Haas and Macdonald, 1999; Bianchi et al., 2002) compared to the depolarizing direction, an effect which is accelerated by neurosteroids (Bianchi and Macdonald, 2003). Although a recent report (Bright et al., 2011) has suggested that the extent of rapid desensitization of this receptor, when recorded at physiological temperature, is much greater than initially reported, there still remains substantial current after rapid application of agonist. Thus, THP's effect to reduce hyperpolarizing current at α4βδ GABARs may be due to desensitization of the steady-state current because it is evident even when agonist is not rapidly applied (Shen et al., 2007). THP-induced desensitization of hyperpolarizing current at α_4_βδ GABARs was shown to be dependent upon the positively charged residue arginine 353 in the TM3-TM4 loop (Shen et al., 2007), which may serve as a modulatory site for Cl^−^. Modulatory effects of Cl^−^ have been reported for other subtypes of GABARs (Olsen and Snowman, 1982; Houston et al., 2009). In contrast, desensitization of the other major extrasynaptic GABAR, α5β3γ2, is accelerated with depolarizing GABAergic current (Burgard et al., 1996).

## Paradoxical excitatory effects of THP on hippocampal function at puberty

The GABAergic current recorded from CA1 hippocampal pyramidal cells in the slice is hyperpolarizing at puberty (Shen et al., 2007). Thus, as would be expected, THP reduces the tonic inhibitory current at puberty (Figure 2), even when action potential-driven GABA release is blocked by tetrodotoxin (Shen et al., 2007), suggesting that it is acting post-synaptically. However, when the direction of the GABAergic current is artificially reversed to depolarizing by increasing intracellular [Cl^−^], THP increases the tonic GABAergic current, suggesting that the effect of the steroid is dependent upon the direction of the Cl^−^ current. As expected, THP increases neuronal excitability at this time, assessed both with cell-attached recordings of spontaneous spiking as well as by current clamp recordings (Shen et al., 2007), which reveal that THP reduces the threshold for spiking by increasing the input resistance of the neuron (Figure 2). In addition, THP administration restores NMDA currents, evoked by low frequency stimulation at puberty, to levels similar to those observed before puberty (Shen et al., 2010a). This latter effect may be due to its reduction of the shunting inhibition produced by α4βδ receptors localized to the dendritic spine at puberty. That possibility is supported by the finding that these paradoxical excitatory effects of the steroid at puberty are not seen in the δ^−/−^ mouse (Shen et al., 2010a), implicating α_4_βδ GABARs. In contrast, THP reduces neuronal excitability before puberty, when α4βδ levels of expression are low.

## Effects of the stress steroid THP on synaptic plasticity and spatial learning at puberty

### Synaptic plasticity

Because THP removes the impediment to activation of NMDA receptors at puberty, additional studies tested whether its administration could also remove the impediment to the induction of LTP at this time, which is dependent upon NMDA receptor activation (Herron et al., 1986). In fact, THP permits robust induction of LTP at puberty, when it is normally not observed (Figure 3), after bath application of the steroid. This outcome is also seen after restricted application of the steroid to the dendrites of the stratum radiatum during theta burst stimulation, suggesting that it facilitates induction (Shen et al., 2010a), rather than maintenance of LTP. This conclusion is confirmed by the finding that THP does not facilitate LTP when it is applied 5 min after theta burst stimulation. THP facilitation of LTP is not seen in the δ^−/−^ hippocampus, suggesting that α4βδ GABARs are responsible for this steroid's effect on synaptic plasticity at puberty (Shen et al., 2010a).

The population of extrasynaptic α5-containing GABARs which are also known to play a role in synaptic plasticity are likely less of a factor in mediating THP's effect. First, they are not reported to localize adjacent to excitatory synapses on spines (Brunig et al., 2002); thus, they would have less of an impact on NMDA receptor activation and the induction of LTP generated by theta burst stimulation. In fact, these receptors have been shown to play a role in frequency-dependent plasticity, such that they lower the frequency necessary to trigger potentiation of CA1 pyramidal cell synaptic responses (Martin et al., 2010). Although it is not yet known whether expression of these receptors increases at the onset of puberty, they are markedly less sensitive to modulation by neurosteroids such as THP compared to α4βδ where THP produces a two-fold increase in potentiation (Belelli et al., 2002). Thus, the small THP-induced potentiation of current at α5-containing GABARs would be expected to only slightly offset the decrease in current at α4βδ GABARs and its effect to trigger robust LTP. The fact that THP completely restores LTP to pre-pubertal levels suggests that any effects of this steroid at α5 GABARs are minor. In fact, the recovery of LTP in the pubertal δ^−/−^ mouse also suggests that the role of α5 GABARs in limiting synaptic plasticity at puberty is also minor.

### Spatial learning

THP also facilitates spatial learning at puberty (Figure 4), assessed using the hippocampal-dependent active avoidance task (Shen et al., 2010a). After THP administration, the pubertal mice reach criterion in less than 3 trials, with a mean latency of 250 s which is more than 3-fold longer than seen in the absence of THP, where mice fail to reach learning criterion in 7 trials. However, THP does not alter the number of shocks delivered per entry, suggesting that pain threshold and other sensorimotor and motivational parameters are not altered by the steroid. Facilitating effects of THP on learning are not seen in the δ^−/−^ mouse, implicating α4βδ GABARs as the target for THP's effect. Because THP can be released by stress (Purdy et al., 1991; Mukai et al., 2008), these results suggest that mild to moderate stress at puberty may have beneficial effects on learning.

## Effects of stress on learning in adolescence

A number of other studies have shown that acute stress can enhance learning during adolescence in rodents (Hodes and Shors, 2005; Uysal et al., 2012). Moderate tailshock improves performance on a trace eyeblink conditioning task administered 24 h later (Hodes and Shors, 2005), an effect independent of the estrous cycle and cortisol levels (Hodes and Shors, 2005). This phenomenon is not seen in pre-pubertal or adult, female rats (Wood and Shors, 1998), suggesting that the pubertal period may be a unique period for stress effects on cognition.

In humans, the effect of stress is complex, and is dependent upon whether the stress is acute or chronic, the degree of stress and the cognitive state of the individual. It also depends upon whether the individual perceives themselves as being in control of life situations (“internal locus of control”) as opposed to feeling a helpless victim of external forces (“external locus of control”). When middle school children were assessed in their academic performance during environmental stressors, those students with an internal locus of control exhibited improved performance during stress (Wolk and Bloom, 1978), while those with an external locus of control had diminished performance as stress level increased. This outcome may also depend on the degree of stress, as first described by the Yerkes-Dodson law (Yerkes and Dodson, 1908), which describes an inverted U relationship between stress and the learning of simple tasks (Lupien et al., 2007).

## Paradoxical anxiety-producing effects of THP at puberty

**Figure 7:**
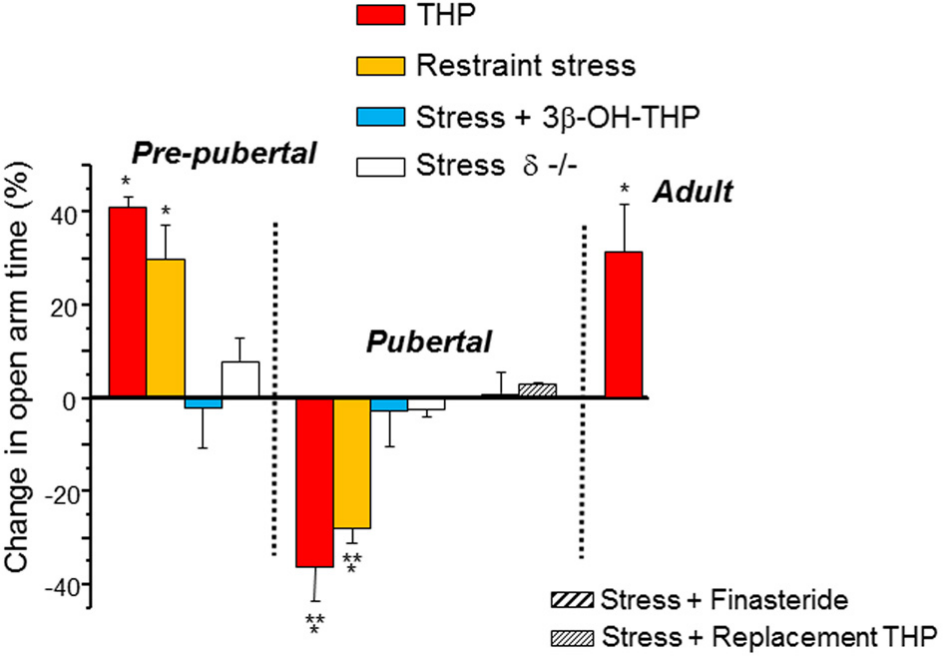
**Paradoxical anxiety-producing effects of THP in pubertal mice**. Changes in anxiety produced by restraint stress or injection of THP (10 mg/kg, i.p.) are represented as a % change in open arm time in the elevated plus maze (EPM) compared to control values. In some cases the inactive 3β-OH isomer of THP (stress + 3β-OH-THP) or finasteride (a 5α-reductase inhibitor) were preadministered to test the role of THP release in the stress response. Replacement THP (10 mg/kg, i.p., in oil, for 3 d) was administered to prevent the decline in THP at puberty. *n* = 6–9 mice for each group. ^*^P = 0.05 vs. control, ^**^P = 0.05 vs. Pre-pub. [Used with permission (Shen et al., 2007)].

GABARs are known to modulate anxiety responses (Rudolph et al., 1999), and are the targets for most anxiety-reducing drugs, including BDZs, barbiturates and alcohol, as well as for THP, which decreases anxiety in adult rodents. Many CNS areas have been implicated in the control of anxiety, including the dentate gyrus (Kheirbek et al., 2013) and hippocampus (Bitran et al., 1999; Bannerman et al., 2004), where direct, local administration of THP can reduce anxiety in adult rats (Bitran et al., 1999; Bannerman et al., 2004). This is consistent with its typical effect to potentiate inhibition at most GABARs (Olsen and Sieghart, 2009). However, THP increases anxiety in pubertal female mice (Figure 6) (Shen et al., 2007) due to the fact that it reduces GABAergic inhibition in the hippocampus at puberty (Shen et al., 2007) via its effect at α4βδ. This was assessed using the elevated plus maze, an animal model of anxiety following direct administration of THP (10 mg/kg, i.p.) and it was also demonstrated indirectly by using 45 min of restraint stress to increase endogenous levels of THP (Higashi et al., 2005), which increases anxiety unless finasteride or the inactive isomer of THP, 3β-OH-THP, is pre-administered to prevent the formation or the effect of THP, respectively. These anxiety-producing effects of THP and restraint stress at puberty are not seen in the δ^−/−^ mouse (Figure 7), implicating α_4_βδ GABARs (Shen et al., 2007). Taken together, these findings suggest that stress-induced release of THP produces anxiety at puberty in contrast to its well-established effect to decrease anxiety at other ages. Although not tested during adolescence, there are other GABAR populations which have been implicated in the anxiety response, including α2-containing and α3-containing GABARs (Rudolph et al., 1999; Atack, 2010, 2011; Smith et al., 2012). Reduced expression of these GABAR subtypes, either by puberty onset alone or by stress during adolescence, would be expected to alter anxiety state.

The paradoxical anxiety-producing effect of THP observed at puberty onset in female mice which is linked with α_4_βδ GABAR expression (Shen et al., 2007) has also been for observed for THP or progesterone, its parent compound, in women with premenstrual dysphoric disorder (Schmidt et al., 1998; Freeman et al., 2002) and menopausal dysphoria (Andreen et al., 2004), when anxiety-related correlates of THP are concentration-dependent.

## Stress and anxiety during adolescence

In humans, anxiety responses to performance stress (mental arithmetic, mirror tracing) and social stress are increased at puberty (Susman et al., 1988; Modesti et al., 1994; Sumter et al., 2010), with a greater prevalence in girls (Garber et al., 2002; Leen-Feldner et al., 2007; Ordaz and Luna, 2012). Anxiety disorders are also most likely to emerge at puberty (Hayward et al., 1992; Costello et al., 2003; Kessler et al., 2005). Brain imaging studies in adolescent girls have correlated increased activity of the limbic system, including hippocampus, with these anxiety responses during a psychosocial stress paradigm (Guyer et al., 2009). Thus, the effect of the stress steroid THP, which reverses at puberty, to increase anxiety, may represent one potential mechanism for these enhanced stress responses in females during adolescence.

## Regulation of α_4_βδ GABAR expression

Recent studies have delineated some of the factors which regulate the expression of α4βδ GABARs. Brain-derived neurotrophic factor (BDNF) plays a role in the activation of the α4 promoter via early growth response factor 3 (Egr3) and the JAK/STAT (Janus kinase/signal transducer and activator of transcription) pathway (Roberts et al., 2006; Lund et al., 2008), as well as in the trafficking of the δ subunit protein to the cell membrane surface (Joshi and Kapur, 2009). Surface expression of α4βδ can be regulated by Ca 2^+^ and extracellular signal-regulated kinase (ERK) 1/2 (Joshi and Kapur, 2013). Expression of α4 can also be induced by heat shock factor 1, which has been shown following exposure of neurons to alcohol (Pignataro et al., 2007).

Hormonal factors which regulate expression of α4βδ GABARs include the ovarian hormone 17β-estradiol (E2), as well as THP. E2, administered either *in vitro* or *in vivo* (Pierson et al., 2005; Shen et al., 2005; Zhou and Smith, 2007) can increase α4 expression in neurons, an effect likely mediated by its ability to increase BDNF (Jezierski and Sohrabji, 2003; Scharfman et al., 2003; Sato et al., 2007). THP, either administered *in vivo* or *in vitro* to cultured neurons, can increase expression of these receptors after 0.5–48 h (Shen et al., 2005; Maguire and Mody, 2007; Kuver et al., 2012). The effect of THP to increase receptor trafficking to the membrane surface is related to its ability to increase the efficacy of the receptor (Kuver et al., 2012), where GABA is a partial agonist (Brown et al., 2002; Bianchi and Macdonald, 2003). GABA alone does not increase receptor expression, but other high efficacy agonists, such as gaboxadol (THIP) and β-alanine, are able to increase surface expression of the receptor, an effect mediated by protein kinase C-δ (Kuver et al., 2012), which has high expression in some of the CNS regions where α4βδ is highly expressed (Messing et al., 2007). [However, protein kinase C-δ does not account for the high expression of α4βδ in other regions, such as dorsal striatrum and nucleus accumbens, which do not have high expression of protein kinase C-δ (Choi et al., 2008)]. This effect of THP to increase surface expression of α4βδ is a result of increases in receptor insertion, rather than through decreases in receptor internalization (Kuver et al., 2012).

Non-hormonal factors which regulate α4βδ expression include alcohol, which reduces expression by activating clathrin-mediated endocytosis (Gonzalez et al., 2012), and increased neuronal excitability produced by neuronal depolarization, NMDA receptor activation, traumatic brain injury or stroke (Payne et al., 2008; Mtchedlishvili et al., 2010; Santhakumar et al., 2010). It is likely that δ-containing GABARs play a neuroprotective role in this regard because excitotoxicity levels are increased in brain tissue from δ^−/−^ animals (Santhakumar et al., 2010).

## Puberty and THP “withdrawal”

THP levels decline by 60–70% at the onset of puberty in both the mouse and the human (Mannan and O'Shaughnessy, 1988; Fadalti et al., 1999; Shen et al., 2007). These declining levels of THP (“THP withdrawal”) appear to be responsible for increases in α4βδ GABAR expression at puberty onset of female mice. First, replacement THP (10 mg/kg, i.p. × 3) during the early days of puberty prevents the increase in α4βδ expression (Shen et al., 2007), as well as the ability of THP to reduce the GABAergic tonic current. Replacement THP also prevents the paradoxical excitatory effects and anxiety-producing effects of THP during adolescence (Shen et al., 2007). Second, a THP withdrawal state induced pharmacologically by administration of the 5α-reductase blocker finasteride, also increases α4βδ GABAR expression in hippocampus of pre-pubertal mice (Smith et al., 2006) and results in paradoxical excitatory effects of THP due to its effect to reduce the tonic inhibitory current.

Withdrawal from progesterone (and thus THP) has also been shown to trigger α4βδ expression in interneurons of the periaqueductal grey (Griffiths and Lovick, 2005; Lovick et al., 2005) on the late diestrous stage of the estrous cycle following the peak in circulating levels of progesterone and THP (Griffiths and Lovick, 2005; Lovick et al., 2005). The resultant increase in tonic inhibition of these interneurons leads to increased excitability of the output neurons, which may represent a mechanism for ovarian cycle-induced panic disorder (Lovick, 2000). In contrast, earlier on diestrus, when circulating levels of progesterone and THP are elevated, expression of α4βδ GABARs is increased on dentate gyrus granule cells in association with an increase in the tonic inhibitory current (Maguire et al., 2005) compared to estrus. Animals at this stage show a decrease in seizure susceptibility as well as decreased anxiety.

THP withdrawal may serve as a hormonal model of premenstrual dysphoric disorder (PMDD), which is characterized by adverse mood, including anxiety, during the decline in circulating levels of progesterone and THP at the end of the luteal phase of the menstrual cycle (Cunningham et al., 2009; Rapkin and Winer, 2009). At this time, stress-triggered anxiety and panic attacks have been reported (Vickers and McNally, 2004; Yonkers et al., 2008; Nillni et al., 2011).

The post-partum period can also be considered a time of “THP withdrawal” when THP levels decline precipitously, and α4βδ expression levels in dentate gyrus granule cells are altered (Maguire and Mody, 2009; Maguire et al., 2009; Sanna et al., 2009). These studies report different outcomes which may be dependent upon the rodent species used (mice vs. rats). In mouse studies (Maguire et al., 2009; Maguire and Mody, 2009), α4βδ expression is decreased in dentate gyrus during pregnancy, as well as in striatrum, but unchanged in cerebral cortex. Expression of these receptors in dentate gyrus then increases during the post-partum period. This change in receptor expression may represent a homeostatic response to maintain normal levels of neuronal excitability. In contrast, in studies using rats (Sanna et al., 2009), δ GABAR expression is increased in dentate gyrus during pregnancy, where these receptors contribute to a greater tonic current. The reason for this discrepancy is not clear but may be due to the different ambient levels of THP which are much higher in mouse brain (Porcu et al., 2010) compared to rat, and therefore suggest that control of α4βδ expression is complex.

## Conclusions

**Figure 8:**
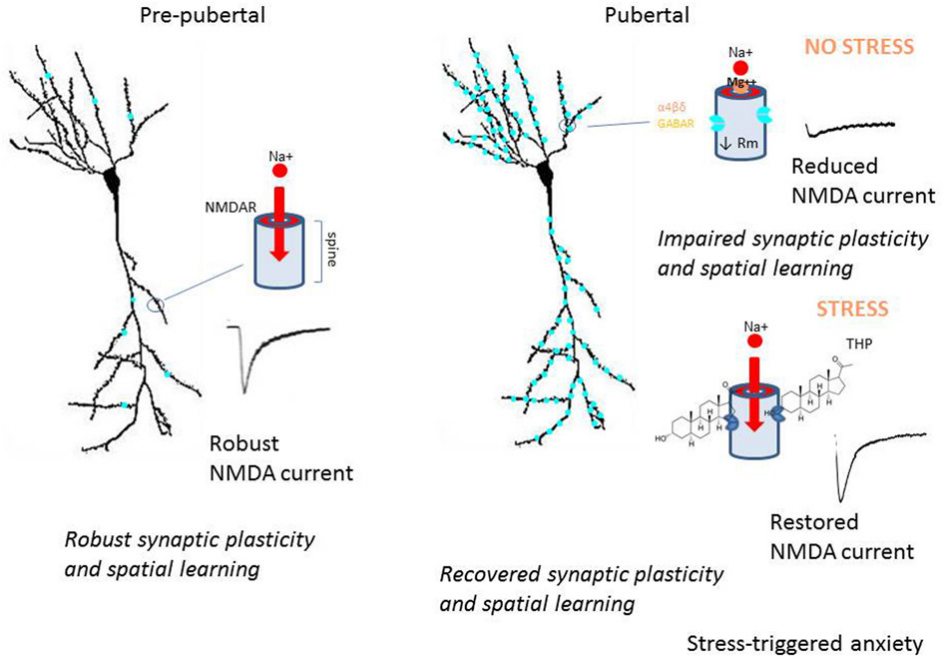
**Summary diagram of pubertal expression of α4βδ GABA_A_ receptors on CA1 pyramidal cells: the impact on neuronal function and behavior**. This summary diagram indicates low expression of α4βδ GABARs (aqua) on CA1 pyramidal cells pre-pubertally (left). During this period, depolarization of the spine permits Mg^2+^ unblock of the NMDA receptor (NMDAR), which allows robust activation of NMDAR and NMDAR-dependent events, such as synaptic plasticity and spatial learning. At puberty (right), α4βδ GABARs increase markedly on both the dendritic shaft and spine, where they shunt depolarizing current (upper diagram). As a result, local depolarization fails to unblock the NMDAR, leading to reduced levels of synaptic plasticity and impaired spatial learning. During sustained stress (lower diagram), release of THP selectively reduces the outward GABA-gated current gated by α4βδ GABARs, thus reducing the shunting tonic inhibition they produce. As a result, NMDAR activation is restored to pre-pubertal levels, as are synaptic plasticity and spatial learning. However, the increase in neuronal excitability produced by this stress steroid is also associated with increases in anxiety behavior. These findings suggest that the emergence of extrasynaptic α4βδ GABARs in the hippocampus at puberty may underlie some of the changes in cognition and mood reported during adolescence.

Hormonally regulated expression of extrasynaptic α4βδ GABARs alters the inhibitory control of neuronal circuits which regulate cognition, seizure threshold and mood (Summary Figure 8). These changes in inhibitory tone are reported across the ovarian cycle, during the post-partum period and at the onset of puberty. Increased expression of α4βδ GABARs during adolescence reduces synaptic plasticity and spatial learning (Summary Figure 8), which may underlie, at least in part, the end of a critical period for optimal learning which has been reported for this time window. In addition, the paradoxical effects of THP at α4βδ GABARs during the pubertal period (Summary Figure 5) may play a role in mood swings and stress-related anxiety which sometimes characterize early adolescence. Genetic aberrations in α4 and/or δ have been reported for certain neuropathologies such as autism, schizophrenia and child-onset mood disorders (Ma et al., 2005; Maldonado-Aviles et al., 2009; Feng et al., 2010). A greater understanding of the role of these extrasynaptic GABARs in behavioral endpoints may help to suggest novel therapeutic strategies for disturbances of mood and cognition.

### Conflict of interest statement

The author declares that the research was conducted in the absence of any commercial or financial relationships that could be construed as a potential conflict of interest.
